# The effect of blood flow-restrictive resistance training on the risk of atherosclerotic cardiovascular disease in middle-aged patients with type 2 diabetes: a randomized controlled trial

**DOI:** 10.3389/fendo.2024.1482985

**Published:** 2024-10-01

**Authors:** Xiaojun Ma, Xuandong Lin, Lei Zhou, Wen Li, Qinyu Yi, Fulian Lei, Xuan Tang, Yuxin Ai, Yating Zhan, Huanyan Luo, Liduo Wang, Fenfang Lei, Binghua He, Fan Yang, Sijie Ruan

**Affiliations:** ^1^ School of Nursing, Shaoyang University, Shaoyang, Hunan, China; ^2^ Department of Conservative Dentistry and Endodontics, College of Stomatology, Guangxi Medical University, Nanning, Guangxi, China; ^3^ Department of Anesthesiology, Central Hospital of Shaoyang, Shaoyang, Hunan, China; ^4^ Department of Endocrinology, The Second Affiliated Hospital of Shaoyang University, Shaoyang, Hunan, China

**Keywords:** blood flow-restrictive resistance training, aerobic exercise, type 2 diabetes mellitus, atherosclerotic cardiovascular disease, risk

## Abstract

**Introduction:**

The aim of this study was to investigate the effects of blood flow-restrictive resistance training (BFR-RT) on improving metabolic abnormalities, blood pressure (BP), obesity, and 10-year atherosclerotic cardiovascular disease (ASCVD) risk in middle-aged patients with type 2 diabetes mellitus (T2DM).

**Method:**

We conducted a parallel-group, single blind randomized controlled trial. Participants who met the inclusion criteria were randomly divided into control group, BFR-RT group and aerobic exercise (AE) group. Control group received health education and follow-up; Two exercise groups received supervised collective training for a period of six months, three times per week. AE group trained at moderate-intensity for 60 minutes each time, while BFR-RT group trained at low-intensity for 40 minutes each time. The primary outcomes were change in 10-year ASCVD risk index and level, and the secondary outcomes included changes in fasting plasma glucose (FPG), glycosylated hemoglobin (HbA1c), blood lipids, BP, and obesity level within and across the three groups at baseline, the third and sixth months of intervention.

**Result:**

Among 93 individuals (control group, n=31; AE, n=30; BFR-RT, n=32) were analyzed. At baseline, there were no significant differences in various indicators among the three groups (p>0.05). After intervention, the 10-year ASCVD risk index and risk level of both exercise groups significantly decreased compared to the control group and baseline (p<0.05), and the risk reduction became more pronounced over time. In the sixth month of intervention, the 10-year ASCVD risk index in the AE group decreased by 27.40%, and that in the BFR-RT group decreased by 26.78%. Meanwhile, apart from lipoprotein (a) and diastolic blood pressure, both exercise groups showed significant improvements in FPG, HbA1c, dyslipidemia, systolic blood pressure, and obesity indicators compared to the control group and baseline (p<0.05). There was no significant difference in various indicators between the two exercise groups (p>0.05).

**Conclusion:**

BFR-RT could reduce the 10-year ASCVD risk in middle-aged T2DM patients for by improving metabolic abnormalities, BP and obesity, and its effect was similar to that of moderate-intensity AE.

**Clinical trial registration:**

https://www.chictr.org.cn/showproj.html?proj=178886, identifier ChiCTR2300074357.

## 1 Introduction

Diabetes shortens the life expectancy of middle-aged people by 4~10 years, and 46.6% of diabetes deaths occur under the age of 60 (1). About 75% of patients with type 2 diabetes mellitus (T2DM) eventually die from cardiovascular diseases (CVD), which is largely due to the uncontrolled risk factors of atherosclerotic cardiovascular disease (ASCVD) (2). Therefore, taking effective measures to reduce the risk of ASCVD in middle-aged T2DM patients plays a crucial role in improving their life years and quality of life.

Patients with T2DM often have hyperglycemia, hypertension, dyslipidemia, obesity, and insulin resistance, which are high-risk factors for ASCVD. Changing unhealthy lifestyle habits is the cornerstone for controlling these risk factors, with exercise being one of the most important measures (3). Numerous studies have shown that moderate exercise can improve metabolic abnormalities in patients with diabetes, enhance cardiorespiratory endurance, and reduce the CVD incidence and all-cause mortality (4). Various guidelines (5) recommend that patients with T2DM engage in at least 150-300 minutes of moderate-intensity or 75-150 minutes of high-intensity aerobic exercise (AE) per week, combined with 2-3 days of moderate-intensity resistance training, to achieve optimal benefits in preventing and controlling cardiovascular complications of T2DM. However, engaging in moderate to high intensity exercise may pose certain challenges for T2DM patients. For instance, some acute or intense exercises may increase the risk of CVD events in middle-aged and elderly individuals with a long-term sedentary lifestyle (6). Some patients may also switch to low-intensity exercise due to factors such as pain, insufficient cardiorespiratory endurance, injury, or fear of accidents (7). Although low-intensity exercise can also help diabetic patients control their metabolic abnormality, it requires longer time commitment and may be less effective (8). As middle-aged individuals experience a decline in physical fitness and face multiple pressures from work, family, and financial constraints, they have less time to allocate for exercise. However, attempting to meet the recommended exercise intensity by increasing the volume of each session and reducing the frequency may increase the risk of exercise-related injuries and CVD events during exercise (9). Therefore, there is an urgent need for a low-time-consuming and effective low-intensity exercise to motivate middle-aged T2DM patients and guide them to gradually adapt to and maintain exercise.

In recent years, blood flow-restrictive resistance training (BFR-RT) has been widely used in the rehabilitation of the elderly, osteoarthritis, and CVD. This low-intensity exercise has attracted attention because it can simulate the muscle mass increase effects of moderate to high-intensity resistance exercise (10). During BFR-RT, an air band is used to apply a certain amount of pressure on the proximal end of the exercising limb, and the limb undergoes resistance training at loads as low as 20% to 40% of an individual’s one-repetition maximum (1-RM, which is the maximum resistance value that can be achieved within the full range of motion of all joints under correct posture and certain rules) under the condition of incomplete occlusion of local blood vessels. The restricted blood flow puts muscle cells in a state of hypoxic stress, leading to the accumulation of metabolic products during exercise, inducing skeletal muscle cell hypertrophy, and improving skeletal muscle strength. With the increase in muscle mass and strength, the body’s basal metabolic rate and glucose demand also increase, which helps maintain the stability of glucose and lipid metabolism and reduces the occurrence of insulin resistance. Su et al. (11) conducted a 12-week BFR-RT on 26 male obese college students, and the results showed that BFR-RT training can effectively improve the body composition of obese college students, increase muscle mass, strength, and endurance, regulate glucose homeostasis, activate neural regulation, and enhance cardiac autonomic regulation. Compared with traditional high-intensity exercise, BFR-RT can benefit the elderly and frail due to its lower cardiovascular and joint pressure. Low-intensity (20% 1-RM) BFR-RT did not induce changes in hemostasis and inflammatory response in patients with stable coronary artery disease and is considered relatively safe (12). Zhuang et al. (13) used lower load resistance exercise (20%–30% 1-RM) combined with 50% of the individual a total limb occlusion pressure (LOP) to restrict blood flow for a 3-month BFR-RT intervention in obese elderly community residents. The experimental results showed that BFR-RT is not only safe for the obese older adults but also helps them increase muscle mass and strength, improve metabolic abnormality, and reduce the risk of sarcopenia and CVD. Therefore, BFR-RT has the potential to become a new exercise intervention measure for controlling cardiovascular complications in diabetes.

However, due to some concerns and controversies, currently few researchers apply BFR-RT to patients with diabetes. Nascimento et al. (14) believe that BFR-RT should be used with caution in diabetic patients, as it may cause sympathetic nerve excitation, increased blood pressure (BP), abnormal cardiovascular responses, and increase the risk of cardiovascular-related adverse events. Lorenz et al. (15) pointed out that these risks are controllable. After adequate medical screening, exercisers wear inflatable cuffs properly, set appropriate inflation pressure and compression time, and the risks generated by BFR-RT are not significantly different from traditional exercise.

Given that most current studies are focused on healthy populations or non-diabetes patients, and the duration of the studies is relatively short, it is still uncertain whether BFR-RT can have a protective effect on the cardiovascular health of middle-aged T2DM patients. Therefore, this study intends to conduct a 6-month exercise intervention on middle-aged T2DM patients to observe whether low-intensity BFR-RT can simulate the effect of moderate-intensity AE in reducing the risk of ASCVD in T2DM patients, aiming to provide new ideas and reference for enriching the diversity of exercise prescription choices for diabetes, and help patients better prevent cardiovascular complications of diabetes.

## 2 Materials and methods

### 2.1 Study design and participants

This study is a single-blind, randomized controlled, parallel trial. After obtaining medical permission from a specialist, all participants were divided into three groups: control group, AE and BFR-RT. They were assigned to a 6-month (24 weeks) exercise programme with three days per week. All participants were evaluated at three time points: before intervention (baseline), the third and sixth months of intervention.

The study period lasted from March 2023 to December 2023. Participants were recruited from the Second Affiliated Hospital of Shaoyang University and the Hongqi Road Community Health Service Center in Shaoyang City through doctor referrals, lectures, and advertisements. The main inclusion criteria were: meeting the WHO diagnostic criteria for T2DM (16), with glycosylated hemoglobin (HbA1c) levels between 6.5% and 11.0%, aged between 40 and 65 years, having a sedentary habit, stable medical conditions and medication use for the three months prior to enrollment, not wearing an insulin pump, clear consciousness, normal comprehension and communication abilities, voluntary participation in the study and signing of an informed consent form. Exclusion criteria included: patients with CVD, such as coronary heart disease (including angina pectoris and myocardial infarction) and stroke; Patients taking weight loss and antipsychotic drugs; Patients has any serious medical condition that would contraindicate for long-term physical activity or unable to complete the exercise programme as required.

To ensure patient safety and the smooth progress of long-term exercise plans, professional exercise rehabilitation specialists assess participants’ exercise risks through the Physical Activity Readiness Questionnaire. Those who pass the test proceed to a 20-minute incremental power cycling test. Researchers record the heart rate, BP, and rating of perceived exertion values of the subjects at the end of each stage during the incremental load test, and analyze data such as metabolic equivalent, reserve oxygen uptake, and heart rate reserve functions to develop personalized exercise plans for the subjects (17). This study has been approved by the Ethics Committee of the Second Affiliated Hospital of Shaoyang University (Ethics No.: 2023-KT037) and has been registered with the China Clinical Trial Registration Center (Registration No.: ChiCTR2300074357). All participants have provided written informed consent, and participants have the right to withdraw from the study at any time for any reason without any consequences. Personal privacy and datasets will be kept confidential.

### 2.2 Randomization and blinding

This study is a single-blind randomized controlled trial. The chief researcher assigns participants a unique code in the order of inclusion to conceal their identity information. Then, a statistician who was not involved in the study stratified the participants based on gender and age in the baseline data, and assigned participants to the control group, AE, and BFR-RT in a 1:1:1 ratio using a computer-generated random sequence to ensure even distribution of participants in each group. The three groups of participants received interventions and assessments in separate locations, with independent researchers responsible for the intervention or follow-up of each group. Data statisticians and all assessment staff were blinded to participant randomization assignment.

### 2.3 Sample size

Higher fasting plasma glucose (FPG) levels are associated with a gradually increasing the risk of ASCVD (18). Therefore, in the pilot study, we tested the mean and standard deviation (mean ± SD) of FPG after intervention: control group, 8.56 ± 1.17; AE, 7.57 ± 1.09; BFR-RT, 7.72 ± 1.14. The sample size was estimated using PASS 15.0 software, and one-way analysis of variance (ANOVA) was used with Alpha = 0.05 and Power = 0.90. To test whether there were significant differences between the three groups in the final data analysis, the total sample size was at least 90, with an average of at least 30 participants per group. Considering a possible 20% attrition rate, 36 participants per group or a total of 108 participants were required.

### 2.4 Intervention measures

#### 2.4.1 Diabetes education

Three groups of participants received the same diabetes health education programme at different times and in different classrooms, taught by the same diabetes specialist nurse. The health education course was 13 hours in total, including 4 hours of offline face-to-face teaching before intervention, and 1.5 hours of online and offline blended teaching once a month during the intervention period. The content was formulated based on the diabetes prevention and control guidelines (19), with the main aim of improving patients’ understanding of diabetes and its complications, exercise, diet, medication, monitoring, and mental health. At the end of each learning session, participants were required to complete relevant knowledge tests to assess their mastery of the knowledge. After class, relevant diabetes health education materials were distributed to participants through WeChat, and questions were answered to ensure they had grasped the relevant knowledge.

#### 2.4.2 Control group

During the study, participants in the control group were encouraged to change their unhealthy lifestyles and cultivate healthy eating and exercise habits based on the content of health education, but we did not provide them with detailed exercise programme and supervised exercise interventions. After obtaining the patients’ consent, researchers recorded their daily exercise, physical energy consumption, and calorie intake through mobile health management software on smartphones. Before the intervention, at the third and sixth months of the intervention, the researchers conducted face-to-face interviews and physical examinations with the participants, mainly to understand their diet, exercise, and changes in their condition, and to provide them with necessary medical assistance and advice.

#### 2.4.3 Exercise intervention

The exercise intervention was implemented in different sports fields. Two weeks before the formal intervention began, participants in the two exercise groups underwent adaptive training. During this period, they were required to fully understand the exercise intervention process and gradually adapt to the exercise intensity. In addition, they also learned how to use the Borg ratings of perceived exertion scale (rating on 6–20), exercise equipment, sports equipment, and possible accident response methods. After conducting a survey on exercise motivation, the researcher arranged the exercise time from 7:30 to 8:30 in the evening. Participants arrived at the corresponding sports field half an hour earlier to receive monitoring of blood glucose, BP, and heart rate. Theirs food intake was enquired by researchers. The training equipment was worn properly by participants. Each sports field had professionally trained coaches and researchers who took turns leading participants in exercise training. During the exercise, participants were required to wear exercise bracelets to monitor heart rate to ensure that participants achieved the target exercise intensity and safety. The training period was six months, three times per week, with an interval of 1-2 days. If participants missed an exercise scheduled, they would make up for it at another time in the same week. There were 15 training sessions per month, and participants were required to have an overall attendance rate of at least 11 times (>70%). If the total attendance rate was still less than 7 times (<50%) after encouragement and communication from the researcher, they were excluded from the final analysis (20).

##### 2.4.3.1 AE group

Participants began their warm-up with low-intensity AE (30% to 39% of heart rate reserve), mainly consisting of simple rhythmic exercises that activated the large joints and muscle groups of the entire body. After the participants adapted, the intensity was increased to moderate (40% to 59% of heart rate reserve) by increasing the speed and frequency of the movements. At this point, the exercisers felt slight sweating and were able to communicate verbally, with an rating of perceived exertion score of 12 to 14 being appropriate (8). Each participant adjusted their movements based on the heart rate displayed in the fitness tracker and their personal physical tolerance to achieve their training goals. The process of each exercise was: 5~10 minutes of warm-up → 45~50 minutes of moderate-intensity AE → 5 minutes of stretching and relaxation, with a total exercise duration of 60 minutes.

##### 2.4.3.2 BFR-RT group

The BFR-RT exercise programme consisted of low-intensity resistance training and limb blood flow restriction. Participants were required to perform resistance training at 20-40% of 1-RM. The researchers estimated the 1-RM values of the upper and lower body muscle strength of individuals through push-ups and leg push tests. After the subjects were familiar with the process and had done sufficient warm-up, they performed the specified movements with an estimated 50% to 70% of individual’s maximum strength as the initial weight, and then gradually increased the resistance until the subjects could not complete the repeated movements. The last successful lift of the weight was the 1-RM value. All repetitive movements should maintain the same speed and range of motion, with a 3-5 minutes interval between each session. The 1-RM value should be obtained within 4 sessions. The subjects were informed of the weight to be used during exercise. The resistance equipment mainly included barbells, elastic ropes, dumbbells, kettlebells, and other small equipment. The movements mainly exercised the large muscle groups of the upper limbs (shoulder press and pull-down, elbow flexion and extension), lower limbs (squat and half squat, leg press), and core (deadlift, hip bridge, plank, crunch). In addition, the researchers selected the KAATSU Air Bands (Product type: C3, USA) suitable for the subject’s limb size and securely tied it to the proximal end of their limb, with a tightness that could accommodate one to two fingers. While gradually inflating the Air Band, the researchers used a laser Doppler flowmeter quantitative analyzer (SONIMAGE, product model: HS1, China) to test the minimum pressure required to block the blood flow in the limb arteries, which is the total limb occlusion pressure (LOP).

**Figure 1:**
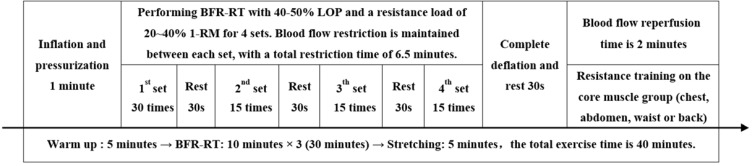
Blood flow-restrictive resistance training scheme. BFR-RT, blood flow-restrictive resistance training; LOP, limb occlusion pressure; 1-RM, one-repetition maximum.

Before starting the BFR-RT, the subjects were required to use the KAATSU to restrict the blood flow in both upper limbs or lower limbs (based on the main exercise muscle group) with 40-50% LOP, while completing four sets of resistance training at 20-40% 1-RM. The exercise volume was 30 repetitions for the first set and 15 repetitions for the second to fourth sets, with a 30-second rest between sets. During this session, the Air Band continued to pressurize and restrict blood flow for 6.5 minutes, then completely deflated to allow blood flow reperfusion for 2 minutes, repeating three times, with a total exercise duration of 30 minutes. The exercisers warmed up for 5 minutes before exercise and stretched for 5 minutes after exercise, with a total exercise duration of 40 minutes. (See **Figure 1** for details).

### 2.5 Outcome measures

The measurement of the outcomes at baseline (month 0), the third and sixth months of the intervention. The demographic and clinical characteristics of the participants were collected by the researchers using a self-designed structured questionnaire. All questionnaires and scales were distributed and collected on-site. The researchers provided necessary explanations to the subjects’ questions, but avoided guiding choices or using suggestive language. After the questionnaires were collected, their completeness and quality were immediately checked. Questionnaires with problems should be confirmed with the subjects again to ensure that the questionnaires were authentic and effective. Blood samples were sent for testing within 2 hours after collection, and the report forms were collected uniformly the next day. Two researchers who did not participate in the group intervention entered the result data into Excel tables, and after verification, they were encrypted and saved. The original examination report was returned to the subjects, and patient privacy was strictly kept confidential.

#### 2.5.1 Primary outcome

The Primary outcome was the change in 10-year ASCVD risk index and risk level. The China-PAR model (21, 22), which is more suitable for Chinese, was selected to predict the risk of cardiovascular and cerebrovascular diseases (including acute myocardial infarction, coronary heart disease death, and stroke) in participants over the next 10 years. The participants’ sex, age, current residence, waist circumference (WC), total cholesterol (TC), high-density lipoprotein cholesterol (HDL-C), current BP level, and whether they have the following conditions: taking antihypertensive drugs, suffering from diabetes, currently smoking, and having a family history of cardiovascular and cerebrovascular diseases (referring to parents, brothers, and sisters who have myocardial infarction or stroke) were all entered into the China-PAR model calculation system (https://www.cvdrisk.com.cn/ASCVD/Eval). The system will automatically provide the subjects’ 10-year ASCVD risk value and stratification: low risk (<5%), moderate risk (5% to 9.9%), or high risk (≥10%).

#### 2.5.2 Secondary outcomes

Secondary outcomes included blood glucose, blood lipids, BP, and obesity indicators. Participants were advised to avoid high-fat foods and alcohol the night before the examination. After fasting for 8-12 hours, they were required to collect venous blood at the designated community health service center at 07:00 am the next morning. Blood samples were centrifuged within 2 hours after collection, and the serum was separated by centrifugation at 3000 rpm for 15 minutes and then frozen at -80°C for analysis of the following indicators: ① The HbA1c level was measured using ion exchange resin high performance liquid chromatography on a Variant II HbA1c analyzer (Bio-Rad, product model: 270-2001, USA). ② FPG, TC, HDL-C, low-density lipoprotein cholesterol (LDL-C), and triglyceride (TG), lipoprotein (a), apolipoprotein A1 (ApoA1), and apolipoprotein B (ApoB) were detected using a Hitachi automatic biochemical analyzer (Hitachi, product model: 7600, Japan). Recent studies (23) have found that interventions that simply increase HDL-C and/or reduce LDL-C do not necessarily reduce the risk of ASCVD, as HDL-C does not fully reflect the function of high density lipoprotein (HDL), and LDL-C is only one of the lipid components that cause atherosclerosis. ApoA1 is the main structural protein of HDL, with stable serum levels, which can better reflect the function of HDL (24). non-HDL-C is the sum of other lipoprotein cholesterol except HDL-C, including LDL-C, intermediate density lipoprotein cholesterol, very low density lipoprotein cholesterol, lipoprotein (a), etc., which covers most of the lipid components that cause ASCVD (25). Each particle of these components contains one ApoB molecule, so ApoB provides a precise estimate of the quantity of atherogenic cholesterol (26). The ApoB/ApoA1 ratio is considered a more convincing marker for evaluating the risk of lipoprotein-related ASCVD (27). Therefore, the ApoB/ApoA1 ratio and non-HDL-C = TC- HDL-C were calculated. Meanwhile, Previous researchers measured FPG and fasting insulin to calculate the insulin resistance index (HOMA-IR), but some patients with diabetes inject insulin, so HOMA-IR may not accurately assess insulin resistance in patients with T2DM. Triglyceride-glucose (TyG) is an easily accessible surrogate marker for insulin resistance. An increase in the TyG index is significantly positively correlated with future risk of ASCVD and the development of T2DM, especially in low- and middle-income countries, where these populations may have a higher susceptibility to insulin resistance (28). When the TyG index is greater than 8.84, the risk of CVD death in pre-diabetes or diabetes patients will increase by 1.77 times (29). Therefore, in this study, TyG was calculated to evaluate the changes in insulin resistance in patients with T2DM. TyG index=Ln [fasting TG (mg/dl) × FPG (mg/dl)/2]. Unit conversion: FPG, 1mmol/L of = 18 mg/dl; TG, 1mmol/L = 88.57mg/dl.

After the subjects rested for 15-30 minutes, their BP was measured. The researchers used an electronic BP monitor (Omron, product model: U730, China) to measure the brachial artery blood pressure of the subjects. The subjects were seated with their arms extended, and the cuff was placed on the upper their arm, with a tightness that could accommodate 1~2 fingers. The lower edge of the cuff was about 2 cm above the strongest point of the brachial artery pulsation in the elbow fossa, so that the BP monitor, brachial artery measurement point, and the heart of the subject were on the same horizontal plane. The subjects remained quiet throughout the measurement process, and the measurement results were accurate to 1 mmHg.

When measuring height, weight, and WC, the subjects should be fasting and empty their bladder. Stand upright on the platform of the electronic scale, with legs together and hands naturally hanging at the sides of the body. After recording the height and weight values, a tape was used to measure the subject’s WC. The weight result is accurate to 1kg, and the height and WC are accurate to 1cm. The waist-to-height ratio (WHtR) = WC (cm)/height (cm), with a WHtR > 0.5 indicating abdominal obesity. The body mass index (BMI) = weight/height^2^ (kg/m^2^), using BMI as a measurement standard (30) to classify the subject’s body shape into: normal (18 to 23.9 kg/m^2^), overweight (24 to 27.9 kg/m^2^), and obese (≥28 kg/m^2^).

#### 2.5.3 Quality control

To reduce the dropout rate of participants, our research group provides regular free physical examinations and professional medical consultations. In collaboration with charitable organizations, we have organized various diabetes-themed activities, including outdoor excursions, knowledge quizzes, and performance reports, with certain rewards given to participants. All researchers have undergone systematic safety training and are familiar with emergency plans for common adverse events during exercise. The adverse events defined in this study refer to unexpected adverse medical events that occur during the participation of subjects in exercise training or research, related to the intensity, frequency, and duration of exercise. Events that lead to serious injury, disability, or loss of function, and even life-threatening events, are defined as serious adverse events. During exercise training or activities, researchers closely monitor the physical condition and reactions of participants, taking necessary preventive and treatment measures in a timely manner to reduce the occurrence of adverse events and minimize their potential harm. At the same time, for adverse events that have already occurred, participants should be required to immediately suspend or terminate the experiment, seek timely medical treatment, and report to the chief researcher to further understand the cause of the event, so as to make necessary compensation and improvements. When rainy or snowy weather is not suitable for outdoor training, we guided participants to practice at home through online live streaming, video release, and other methods.

### 2.6 Statistical analysis

Statistical analysis of the data was performed using SPSS 23.0 (SPSS Inc., Chicago, IL, USA). When analyzing the baseline data of the three groups, ANOVA or Kruskal-Wallis test was used for the comparison of measurement data, and the chi-square test was used for the comparison of count data. If more than 20% of the cells have an expected count less than 5, the result of Fisher’s exact test is used. The independent sample t-test was used to compare the attendance rates of the two exercise groups. Two-way repeated measures analysis of variance was used to compare whether there were statistical differences in the dependent variables of the three groups at the three time points. When the results of repeated measures analysis of variance do not meet Mauchly’s test of sphericity, the test result is based on Greenhouse-Geisser. The Bonferroni test was used for multiple comparison of means after the event. The mean difference (95% IC) was used to indicate the mean difference within the group from the third months (T3) or sixth months (T6) of intervention to the baseline (T0). The effect size of repeated measures analysis of variance is represented by partial Eta squared (η^2^P: small ≥ 0.01, medium ≥ 0.06, large ≥ 0.14). The effect size of the mean difference between groups is represented by Cohen’s d (d: small ≥ 0.2, medium ≥ 0.5, large ≥ 0.8). The level of statistical significance is defined as 0.05.

## 3 Results

### 3.1 Participant registration, allocation, and drop-out rate

**Figure 2:**
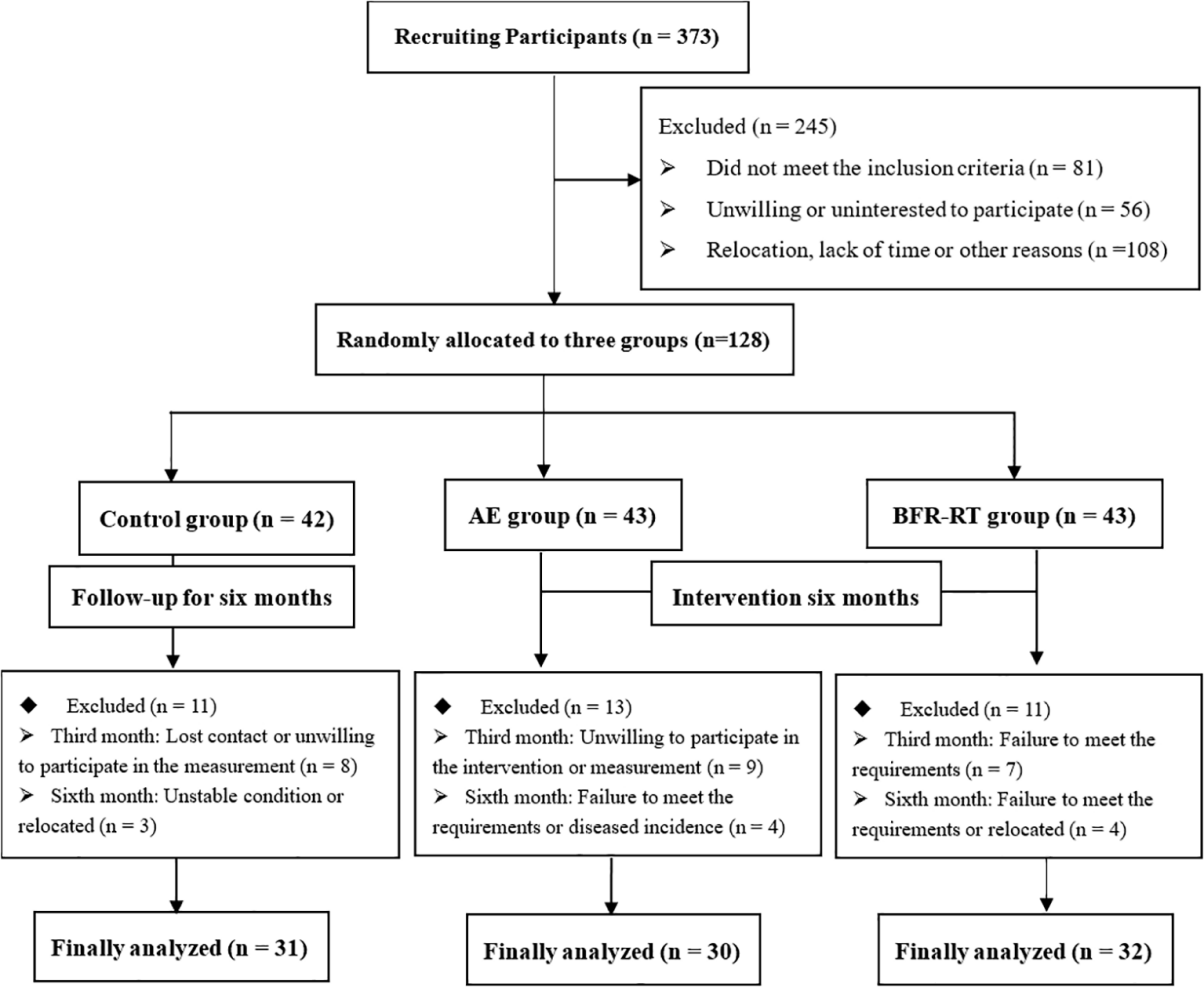
Flow chart of participant enrollment, allocation, and analysis.

**Figure 2** shows that among the 373 patients of T2DM who initially participated in the screening, a total of 128 patients entered the random allocation. During the study, 35 participants were excluded due to loss of contact, unwillingness to continue participating in physical examinations or exercise interventions, failure to meet requirements, relocation, and diseases. In the end, a total of 93 participants completed the 6-month intervention or follow-up. The control group had 31 participants (drop-out rate of 26.19%), AE group had 30 participants (drop-out rate of 30.23%), and BFR-RT group had 32 participants (drop-out rate of 25.58%) whose data entered the final analysis.

### 3.2 Participant demographics and clinical characteristics

**Table 1** shows the demographic and clinical characteristics of the three groups of participants at baseline. There were no significant differences in baseline comparisons between the three groups (p > 0.05). The mean age of the 93 participants was 57.33 ± 5.81, with 43 males (46.2%) and 50 females (53.8%). 72 participants (77.42%) had abdominal obesity, 67 participants (72.04%) were overweight, and 16 participants (17.20%) were obese. All participants were from urban (74, 79.6%) or rural (19, 20.4%) areas of Daxiang District, Shaoyang City, Hunan Province (located in southern China). They had been diagnosed with T2DM for at least 2 years, and there were no changes in medication method, medication type, or smoking habits during the intervention period. However, a small number of participants adjusted the dose of their hypoglycemic drugs due to changes in their condition, with 2 participants in the control group increasing the dose, 2 participants in the AE group decreasing the dose, and 3 participants in the BFR-RT group decreasing the dose. The difference in medication dose adjustments between the three groups was not significant (χ²=5.373, p=0.202). The attendance rate in the AE group was (82.22 ± 2.47)% and the BFR-RT group was (81.96 ± 2.43)%. There was no significant difference in attendance rates between the two exercise groups (t=0.416, p=0.679).

**Table 1:** Baseline demographic and clinical characteristics for the three groups (mean ± SD/n, %).

Characteristic		Group (n=93)			*F*/χ^2^	*p*
		Control (n=31)	AE (n=30)	BFR-RT (n=32)		
Age, years		56.29 ± 5.92	57.73 ± 5.85	57.56 ± 4.85	0.622	0.539
Height, cm		163.61 ± 7.18	165.53 ± 8.90	164.59 ± 9.35	0.386	0.681
10 years of ASCVD risk index,%		8.60 ± 2.62	8.54 ± 2.77	8.29 ± 3.29	0.100	0.905
Sex	Male	11(35.5)	16 (53.3)	16 (50.0)	2.232	0.328
	Female	20 (64.5)	14 (46.7)	16 (50.0)		
Current residence	City	23 (74.2)	25 (83.3)	26 (81.2)	0.868	0.648
	Countryside	8 (25.8)	5 (16.7)	6 (18.8)		
Medications used for blood pressure	Yes	23 (74.2)	19 (63.3)	21 (65.6)	0.923	0.630
	No	8 (25.8)	11 (36.7)	11 (34.4)		
Current smoking	Yes	9 (29.0)	12 (40.0)	12 (37.5)	0.888	0.642
	No	22 (71.0)	18 (60.0)	20 (62.5)		
CVD family history	Yes	11 (35.5)	8 (26.7)	14 (43.8)	1.974	0.373
	No	20 (64.5)	22 (73.3)	18 (56.2)		
Course of diabetes, years	2–5	13 (41.9)	15 (50.0)	14 (43.8)	0.961	0.916
	5–10	11 (35.5)	10 (33.3)	13 (40.6)		
	>10	7 (22.6)	5 (16.7)	5 (15.6)		
Glucose-lowering medication	Oral medication	17 (54.8)	14 (46.7)	19 (59.3)	3.229	0.520
	Oral medication + insulin	6 (19.4)	11 (36.7)	7 (21.9)		
	Other	8 (25.8)	5 (16.6)	6 (18.8)		
Medications used for dyslipidemia	Yes	13 (41.9)	10 (33.3)	8 (25.0)	2.032	0.362
	No	18 (58.1)	20 (66.7)	24 (75.0)		
Blood pressure	Systolic, mmHg	132.48 ± 10.19	130.67 ± 9.00	131.31 ± 11.15	0.251	0.779
	Diastolic,mmHg	79.06 ± 8.79	80.13 ± 8.60	78.50 ± 9.24	0.268	0.765
Diabetes indicators	FPG, mmol/L	8.36 ± 1.13	8.11 ± 1.30	8.18 ± 1.36	0.337	0.715
	HbA1c,%	8.23 ± 1.02	7.87 ± 0.93	8.01 ± 0.83	1.134	0.326
Blood lipid components	TC, mmol/L	4.58 ± 0.96	4.36 ± 0.78	4.67 ± 0.75	1.121	0.330
	TG, mmol/L	2.11 ± 1.04	1.84 ± 1.03	1.95 ± 0.84	0.602	0.550
	LDL-C, mmol/L	2.63 ± 0.64	2.55 ± 0.74	2.69 ± 0.65	0.346	0.708
	HDL-C,mmol/L	1.01 ± 0.32	1.01 ± 0.21	1.10 ± 0.24	1.310	0.275
	non- HDL-C, mmol/L	3.57 ± 0.92	3.34 ± 0.73	3.57 ± 0.83	1.674	0.512
	lipoprotein (a), mg/L	111.58 ± 45.32	115.60 ± 46.06	110.03 ± 43.22	0.126	0.882
	ApoA1, g/L	1.16 ± 0.37	1.09 ± 0.31	1.17 ± 0.20	0.672	0.513
	ApoB, g/L	0.84 ± 0.25	0.82 ± 0.19	0.86 ± 0.22	0.375	0.688
	ApoB/ApoA1	0.78 ± 0.30	0.81 ± 0.32	0.76 ± 0.22	0.263	0.770
Obesity indicators	TyG index	9.44 ± 0.52	9.23 ± 0.57	9.36 ± 0.46	1.192	0.308
	WC, cm	88.94 ± 8.12	88.80 ± 7.72	86.03 ± 9.22	1.201	0.306
	Weight, kg	72.32 ± 8.93	73.07 ± 9.52	71.63 ± 10.11	0.177	0.838
	BMI, kg/m^2^	26.92 ± 1.74	26.56 ± 1.55	26.33 ± 1.76	0.984	0.378
	WHtR	0.54 ± 0.04	0.54 ± 0.04	0.52 ± 0.05	1.817	0.168

AE, aerobic exercise group; BFR-RT, blood flow-restrictive resistance training group; FPG, fasting plasma glucose; HbA1c, Glycosylated hemoglobin; TC, total cholesterol; TG, triglycerides; HDL-C, high-density lipoprotein cholesterol; LDL-C, low-density lipoprotein cholesterol; WHtR, waist-to-height ratio; BMI, body mass index; ApoA1, ApolipoproteinA1; ApoB, ApolipoproteinB; TyG, triglyceride-glucose index; WC, waist circumference.

### 3.3 Results of repeated analysis of variance

**Tables 2**–**6** report the results of repeated measures analysis of variance for 10-year ASCVD risk index, glucose, lipids, BP, and anthropometric indicators used to evaluate the three groups. The Shapiro-Wilk test showed that all dependent variables were basically normally distributed (p > 0.05). The Mauchly’s test of sphericity results showed that HbA1c, TG, LDL-C, HDL-C, ApoB, TyG and systolic blood pressure (SBP) conformed to the sphericity test hypothesis (p > 0.05), while the remaining dependent variables did not conform to the sphericity test hypothesis (p < 0.05). The Levene test indicated that the variance of each dependent variable was homogeneous (p > 0.05). Among all the results of repeated measures analysis of variance, except for the main effect of group on lipoprotein (a) and diastolic blood pressure (DBP) (p > 0.05), all other dependent variables were significantly affected by the interaction effect of (group × time), the main effects of group and time (p < 0.05).

**Table 2:** Effects of interventions on 10 years ASCVD risk index among the three groups (mean ± SD or mean difference [95% CI]).

Index and time points	Group (n=93)			Cohen’s d			Repeated measurement analysis of variance			
	Control (n=31)	AE (n=30)	BFR-RT (n=32)	AE versus Control	BFR-RT versus Control	BFR-RT versus AE		Group × Time Interaction	Time	Group
10 years atherosclerotic cardiovascular disease (ASCVD) risk index, %										
T0	8.60 ± 2.62	8.54 ± 2.77	8.29 ± 3.29	-0.022	-0.104	-0.082	*F*	13.653	55.418	4.017
T3	8.78 ± 2.58	7.17 ± 2.29	6.90 ± 2.36	-0.659*	-0.761*	-0.116	p	<0.001	<0.001	0.021
T6	8.55 ± 1.97	6.19 ± 1.88	6.07 ± 2.28	-1.225#	-1.163#	-0.057	η^2^P	0.233	0.381	0.082
T3 versus T0	0.18(-0.47, 0.83)	-1.37(-2.03, -0.71)#	-1.39(-2.03,-0.75)#							
T6 versus T0	-0.05(-0.75, 0.65)	-2.34(-3.05, -1.63)#	-2.22(-2.91, -1.54)#							

AE, aerobic exercise group; BFR-RT, blood flow-restrictive resistance training group; η^2^P, partial eta-squared; T0, baseline; T3, at third month; T6, at sixth month; *, significant difference at p < 0.05; #, significant difference at p < 0.001.

**Table 3:** Effects of interventions on FPG and HbA1c among the three groups (mean ± SD or mean difference [95% CI]).

Index and time points	Group (n=93)			Cohen’s d			Repeated measurement analysis of variance			
	Control (n=31)	AE (n=30)	BFR-RT (n=32)	AE versus Control	BFR-RT versus Control	BFR-RT versus AE		Group × Time Interaction	Time	Group
Fasting plasma glucose (FPG), mmol/L										
T0	8.36 ± 1.13	8.11 ± 1.30	8.18 ± 1.36	-0.206	-0.144	0.053	*F*	4.663	8.173	3.220
T3	8.53 ± 1.28	7.77 ± 1.11	7.80 ± 1.11	-0.634*	-0.610*	0.027	p	0.002	0.001	0.045
T6	8.48 ± 1.08	7.58 ± 0.99	7.72 ± 0.89	-0.868*	-0.769*	0.149	η^2^P	0.094	0.083	0.067
T3 versus T0	0.17(-0.11, 0.44)	-0.33(-0.62, 0.05)*	-0.37(-0.65, -0.10)*							
T6 versus T0	0.11(-0.24, 0.47)	-0.53(-0.89, -0.16)*	-0.46(-0.81, -0.10)*							
Glycosylated hemoglobin (HbA1c), %										
T0	8.23 ± 1.02	7.87 ± 0.93	8.01 ± 0.83	-0.369	-0.237	0.159	*F*	16.495	92.671	3.706
T3	8.22 ± 0.97	7.55 ± 0.91	7.73 ± 0.88	-0.712*	-0.530	0.201	p	<0.001	<0.001	0.028
T6	8.17 ± 0.97	7.35 ± 0.84	7.57 ± 0.89	-0.903*	-0.645*	0.254	η^2^P	0.268	0.507	0.076
T3 versus T0	-0.01(-0.11, 0.10)	-0.32(-0.43, -0.21)#	-0.28(-0.39, -0.17)#							
T6 versus T0	-0.06(-0.18, 0.06)	-0.52(-0.64, -0.40)#	-0.44(-0.55, -0.32)#							

BFRE, blood flow-restrictive resistance exercise group; RT, moderate-intensity resistance training group; η^2^P, partial eta-squared; T0, baseline; T3, at third month; T6, at sixth month; *, significant difference at p < 0.05; #, significant difference at p < 0.001.

**Table 4:** Effects of interventions on blood lipid profile among the three groups (mean ± SD or mean difference [95% CI]).

Index and time points	Group (n=93)			Cohen’s d			Repeated measurement analysis of variance			
	Control (n=31)	AE (n=30)	BFR-RT (n=32)	AE versus Control	BFR-RT versus Control	BFR-RT versus AE		Group × Time Interaction	Time	Group
Total cholesterol (TC), mmol/L										
T0	4.58 ± 0.96	4.36 ± 0.78	4.67 ± 0.75	-0.251	0.105	0.405	*F*	15.532	26.162	3.200
T3	4.76 ± 0.97	4.17 ± 0.75	4.33 ± 0.62	-0.679*	-0.530	0.233	p	<0.001	<0.001	0.045
T6	4.68 ± 0.92	4.01 ± 0.73	4.11 ± 0.65	-0.805*	-0.718*	0.145	η^2^P	0.257	0.225	0.066
T3 versus T0	0.19(0.04, 0.33)*	-0.20(-0.34, -0.05)*	-0.34(-0.48, -0.20)#							
T6 versus T0	0.11(-0.08, 0.29)	-0.36(-0.54, 0.17)#	-0.56(-0.74, -0.38)#							
Triglyceride (TG), mmol/L										
T0	2.11 ± 1.04	1.84 ± 1.03	1.95 ± 0.84	-0.261	-0.170	0.117	*F*	9.371	12.764	3.147
T3	2.19 ± 0.83	1.62 ± 0.97	1.75 ± 0.73	-0.632*	-0.564	0.152	p	<0.001	<0.001	0.048
T6	2.24 ± 0.93	1.53 ± 0.90	1.59 ± 0.52	-0.776*	-0.866*	0.082	η^2^P	0.172	0.124	0.065
T3 versus T0	0.07(-0.07, 0.22)	-0.22(-0.37, -0.07)*	-0.20(-0.34, -0.06)*							
T6 versus T0	0.13(-0.04, 0.29)	-0.31(-0.48, -0.14)*	-0.36(-0.52, -0.19)#							
Low density lipoprotein cholesterol (LDL-C), mmol/L										
T0	2.63 ± 0.64	2.55 ± 0.74	2.69 ± 0.65	-0.116	0.093	0.201	*F*	40.820	86.195	3.516
T3	2.76 ± 0.63	2.26 ± 0.74	2.33 ± 0.59	-0.729*	-0.705*	0.105	p	<0.001	<0.001	0.034
T6	2.73 ± 0.69	2.05 ± 0.72	2.12 ± 0.59	-0.965#	-0.951*	0.107	η^2^P	0.476	0.489	0.072
T3 versus T0	0.14(0.03, 0.25)*	-0.29(-0.40, -0.18)#	-0.37(-0.47, -0.26)#							
T6 versus T0	0.11(-0.01, 0.22)	-0.50(-0.62, -0.39)#	-0.57(-0.68, -0.46)#							
High density lipoprotein cholesterol (HDL-C), mmol/L										
T0	1.01 ± 0.32	1.01 ± 0.21	1.10 ± 0.24	-0.017	0.319	0.398	*F*	5.079	109.128	4.555
T3	1.14 ± 0.24	1.17 ± 0.23	1.28 ± 0.31	0.128	0.504	0.401	p	0.001	<0.001	0.013
T6	1.22 ± 0.30	1.44 ± 0.28	1.53 ± 0.28	0.758*	1.069#	0.321	η^2^P	0.101	0.548	0.092
T3 versus T0	0.13(0.03, 0.23)*	0.16(0.06, 0.27)#	0.18(0.08, 0.28)#							
T6 versus T0	0.21(0.10, 0.32)#	0.43(0.32, 0.55)#	0.42(0.32, 0.53)#							
non-High density lipoprotein cholesterol (non-HDL-C), mmol/L										
T0	3.57 ± 0.92	3.36 ± 0.73	3.57 ± 0.83	-0.252	0.007	0.268	*F*	18.097	107.651	4.786
T3	3.63 ± 1.01	3.00 ± 0.70	3.05 ± 0.73	-0.723*	0.660*	0.070	p	<0.001	<0.001	0.011
T6	3.43 ± 0.85	2.57 ± 0.70	2.59 ± 0.73	-1.103#	-1.062#	0.028	η^2^P	0.287	0.545	0.096
T3 versus T0	0.06(-0.10, 0.22)	-0.36(-0.51, -0.20)#	-0.52(-0.68, -0.37)#							
T6 versus T0	-0.14(-0.34, 0.07)	-0.79(-1.00, -0.58)#	-0.99(-1.19, -0.78)#							
Lipoprotein (a) [Lp (a)], mg/L										
T0	111.58 ± 45.32	115.60 ± 46.06	110.03 ± 43.22	0.088	-0.035	-0.125	*F*	6.067	9.307	0.259
T3	112.95 ± 45.23	116.64 ± 44.02	105.44 ± 40.69	0.083	-0.175	-0.265	p	<0.001	<0.001	0.773
T6	114.09 ± 45.71	108.93 ± 44.77	101.34 ± 43.61	-0.114	-0.286	-0.172	η^2^P	0.119	0.094	0.006
T3 versus T0	1.37(-3.24, 5.99)	-3.96(-8.65, 0.73)	-4.59(-9.13, -0.05)*							
T6 versus T0	2.51(-0.81, 5.83)	-6.68(-10.05, -3.30)#	-8.69(-11.96, -5.43)#							
ApolipoproteinA1 (ApoA1), g/L										
T0	1.16 ± 0.37	1.09 ± 0.31	1.17 ± 0.20	-0.205	0.034	0.309	*F*	29.663	99.332	4.028
T3	1.12 ± 0.33	1.24 ± 0.33	1.35 ± 0.19	0.364	0.858*	0.412	p	<0.001	<0.001	0.021
T6	1.15 ± 0.36	1.37 ± 0.32	1.51 ± 0.20	0.645*	1.242#	0.529	η^2^P	0.397	0.525	0.082
T3 versus T0	-0.04(-0.09, 0.01)	0.15(0.09, 0.20)#	0.17(0.12, 0.22)#							
T6 versus T0	-0.01(-0.08, 0.06)	0.28(0.21, 0.34)#	0.34(0.27, 0.41)#							
ApolipoproteinB (ApoB), g/L										
T0	0.84 ± 0.25	0.82 ± 0.19	0.86 ± 0.22	-0.090	0.085	0.194	*F*	30.354	4.740	3.207
T3	0.91 ± 0.22	0.75 ± 0.17	0.80 ± 0.18	-0.812*	-0.548	0.285	p	<0.001	0.010	0.045
T6	0.94 ± 0.25	0.74 ± 0.16	0.76 ± 0.18	-0.950*	-0.828*	0.117	η^2^P	0.403	0.050	0.067
T3 versus T0	0.07(0.03, 0.11)#	-0.06(-0.10, -0.03)#	-0.06(-0.10, -0.03)#							
T6 versus T0	0.11(0.07, 0.14)#	-0.08(-0.11, -0.04)#	-0.11(-0.14, -0.08)#							
ApoB/ApoA1 ratio										
T0	0.78 ± 0.30	0.81 ± 0.32	0.76 ± 0.22	0.097	-0.076	-0.183	*F*	37.731	46.815	7.405
T3	0.87 ± 0.29	0.66 ± 0.28	0.60 ± 0.14	-0.737*	-1.192#	-0.274	p	<0.001	<0.001	0.001
T6	0.88 ± 0.28	0.57 ± 0.17	0.50 ± 0.11	-1.333#	-1.797#	-0.492	η^2^P	0.456	0.342	0.141
T3 versus T0	0.09(0.04, 0.14)#	-0.15(-0.20, -0.10)#	-0.15(-0.20, -0.10)#							
T6 versus T0	0.10(0.03, 0.17)#	-0.24(-0.31, -0.17)#	-0.25(-0.32, -0.19)#							

AE, aerobic exercise group; BFR-RT, blood flow-restrictive resistance training group; η^2^P, partial eta-squared; T0, baseline; T3, at third month; T6, at sixth month; *, significant difference at p < 0.05; #, significant difference at p < 0.001.

**Table 5:** Effects of interventions on blood pressure values among the three groups (mean ± SD or mean difference [95% CI]).

Index and time points	Group (n=93)			Cohen’s d			Repeated measurement analysis of variance			
	Control (n=31)	AE (n=30)	BFR-RT (n=32)	AE versus Control	BFR-RT versus Control	BFR-RT versus AE		Group × Time Interaction	Time	Group
Systolic blood pressure (SBP), mmHg										
T0	132.48 ± 10.19	130.67 ± 9.00	131.31 ± 11.15	-0.188	-0.109	0.063	*F*	7.616	4.416	3.679
T3	134.81 ± 7.56	127.33 ± 9.10	128.84 ± 10.58	-0.896*	-0.648*	0.153	p	<0.001	0.013	0.029
T6	135.06 ± 8.73	126.03 ± 9.91	128.16 ± 11.30	-0.968*	-0.682*	0.200	η^2^P	0.145	0.047	0.076
T3 versus T0	2.32(-0.20, 4.85)	-3.33(-5.90, -0.77)*	-2.47(-4.96, -0.02)*							
T6 versus T0	2.58(-0.18, 5.34)	-4.63(-7.44, -1.83)#	-3.16(-5.87, -0.44)*							
Diastolic blood pressure (DBP), mmHg										
T0	79.06 ± 8.79	80.13 ± 8.60	78.34 ± 8.99	0.123	-0.081	-0.203	*F*	1.721	5.474	0.748
T3	79.87 ± 7.51	78.80 ± 7.92	76.78 ± 7.70	-0.139	-0.406	-0.259	p	0.154	0.006	0.476
T6	79.03 ± 6.79	77.23 ± 7.92	76.16 ± 7.79	-0244	-0.392	-0.136	η^2^P	0.037	0.057	0.016
T3 versus T0	0.81(-1.37, 2.97)	-1.33(-3.55, -0.88)	-1.56(-3.70, -0.58)							
T6 versus T0	-0.03(-2.51, 2.45)	-2.90(-5.42, -0.38)*	-2.19(-4.63, -0.25)							

AE, aerobic exercise group; BFR-RT, blood flow-restrictive resistance training group; η^2^P, partial eta-squared; T0, baseline; T3, at third month; T6, at sixth month; *, significant difference at p < 0.05; #, significant difference at p < 0.001.

**Table 6:** Effects of interventions on WHtR, BMI and TyG among the three groups (mean ± SD or mean difference [95% CI]).

Index and time points	Group (n=93)			Cohen’s d			Repeated measurement analysis of variance			
	Control (n=31)	AE (n=30)	BFR-RT (n=32)	AE versus Control	BFR-RT versus Control	BFR-RT versus AE		Group × Time Interaction	Time	Group
Waist-to-height ratio (WHtR)										
T0	0.54 ± 0.04	0.54 ± 0.04	0.52 ± 0.05	-0.178	-0.441	-0.440	*F*	154.358	216.446	9.346
T3	0.55 ± 0.04	0.52 ± 0.03	0.50 ± 0.05	-0.847*	-1.102#	-0.481	p	<0.001	<0.001	<0.001
T6	0.56 ± 0.04	0.49 ± 0.04	0.49 ± 0.05	-1.750#	-1.543#	-0.073	η^2^P	0.774	0.706	0.172
T3 versus T0	0.01(0.002, 0.01)*	-0.02(-0.02, -0.02)#	-0.02(-0.02, -0.02)#							
T6 versus T0	0.01(0.01, 0.02)#	-0.04(-0.05, -0.04)#	-0.03(-0.04, -0.03)#							
Body mass index (BMI), kg/m^2^										
T0	26.92 ± 1.74	26.56 ± 1.55	26.32 ± 1.76	-0.218	-0.343	-0.144	*F*	123.288	178.80	10.768
T3	27.28 ± 1.72	25.44 ± 1.39	25.62 ± 1.72	-1.175#	-0.965#	0.155	p	<0.001	<0.001	<0.001
T6	27.49 ± 1.73	24.76 ± 1.40	24.87 ± 1.51	-1.732#	-1.615#	0.075	η^2^P	0.733	0.665	0.193
T3 versus T0	0.36(0.18,0.53)#	-1.13(-1.31, -0.95)#	-0.71(-0.88, -0.53)#							
T6 versus T0	0.57(0.32, 0.82)#	-1.81(-2.06, -1.55)#	-1.46(-1.70, -1.21)#							
Triglyceride-glucose index (TyG)										
T0	9.44 ± 0.52	9.23 ± 0.57	9.36 ± 0.46	-0.385	-0.163	0.252	*F*	17.844	19.805	5.852
T3	9.52 ± 0.44	9.05 ± 0.59	9.21 ± 0.42	-0.905*	-0.721*	0.314	p	<0.001	<0.001	0.004
T6	9.54 ± 0.43	8.98 ± 0.55	9.14 ± 0.38	-1.137#	0.987#	0.340	η^2^P	0.284	0.180	0.115
T3 versus T0	0.08(-0.004, 0.17)	-0.18(-0.26, -0.10)#	-0.14(-0.23, -0.07)#							
T6 versus T0	0.11(-0.01, 0.20)*	-0.25(-0.34, -0.16)#	-0.22(-0.31, 0.13)#							

AE, aerobic exercise group; BFR-RT, blood flow-restrictive resistance training group; η^2^P, partial eta-squared; T0, baseline; T3, at third month; T6, at sixth month; *, significant difference at p < 0.05; #, significant difference at p < 0.001.

### 3.4 10-year ASCVD risk

**Figure 3:**
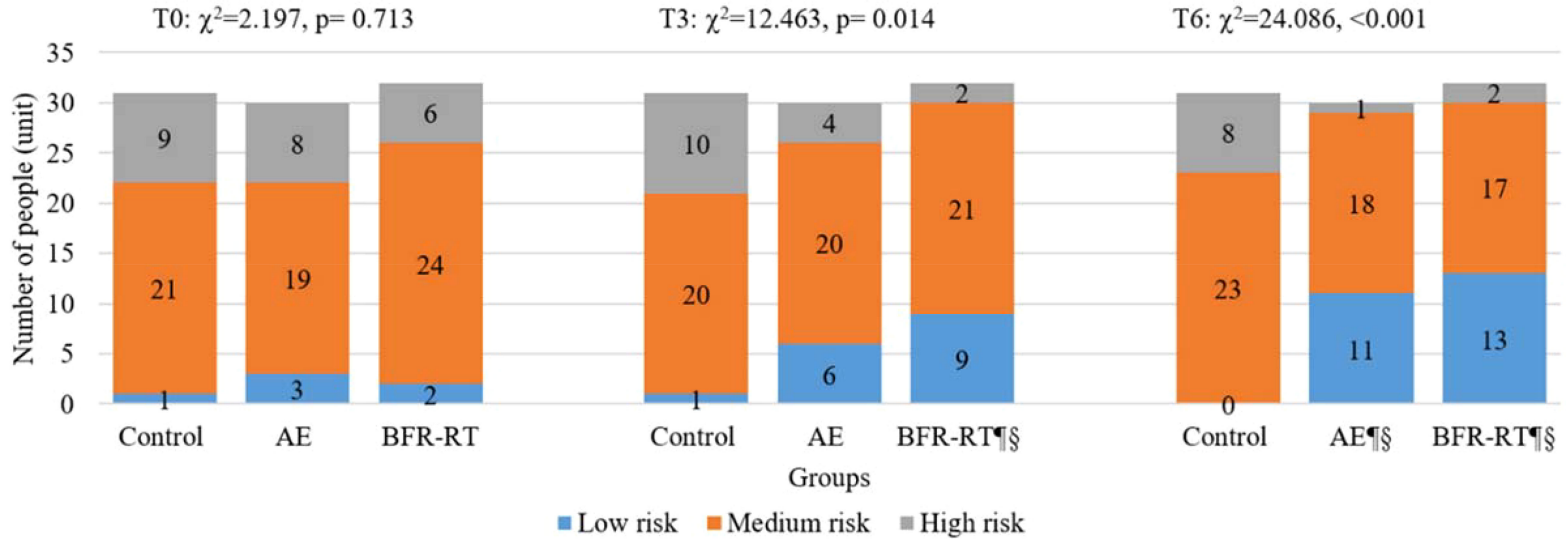
Changes in 10-year ASCVD risk levels for three groups. AE, aerobic exercise group; BFR-RT, blood flow-restrictive resistance training group; T0, baseline; T3, at third month; T6, at sixth month; ¶, significant difference compared to baseline, p < 0.05; §, significant difference compared to control group, p < 0.05.

**Figure 3** reports the changes in 10-year ASCVD risk levels among the three groups of participants before and after the intervention. Before the intervention, 68.82% of the participants were at moderate risk, 24.73% were at high risk, and only 6.45% belonged to the low-risk for 10-year ASCVD risk. At baseline, there was no significant difference in the 10-year ASCVD risk levels among the three groups of participants (χ^χ²^=2.197, p=0.713). In the third month of intervention, there was a significant difference in the 10-year ASCVD risk levels among the three groups (χ^2^ = 12.463, p=0.014), with BFR-RT group showing a significant difference compared to the control group (χ^2^ = 11.836, p=0.003) and baseline(χ^2^ = 6.479, p=0.039), and AE group showing no significant difference compared to BFR-RT group and the control group (p>0.05). In the sixth month of the intervention, there was a significant difference in the 10-year ASCVD risk levels among the three groups (χ²=24.086, p<0.001). The 10-year ASCVD risk levels of both exercise groups were significantly lower than those of the control group(p<0.001) and baseline (p<0.05).

**Table 2** shows the changes in 10-year ASCVD risk index for the three groups. At baseline, there was no significant difference in the 10-year ASCVD risk index among the three groups (F=0.100, p=0.905). From the third month of intervention, the 10-year ASCVD risk index for both exercise groups had significantly decreased compared to the baseline and the control group (p<0.05). By the sixth month of intervention, the effects of both exercises groups on reducing the 10-year ASCVD risk in patients with T2DM were more pronounced (p<0.001). The 10-year ASCVD risk index in the AE group decreased, with a mean (95% CI) change of-2.34(-3.05, -1.63), a decrease of 27.40%, while the BFR-RT group decreased by -2.22(-2.91, -1.54), a decrease of 26.78%. However, there was no significant change in the 10-year ASCVD risk index and level for the control group before and after the intervention (p>0.05). There was no significant difference in the 10-year ASCVD risk index and levels between the two exercise groups (p>0.05).

### 3.5 FPG and HbA1c

**Table 3** shows the changes in FPG and HbA1c among the three groups. There were no significant differences in FPG and HbA1c among the three groups at baseline (p > 0.05). Compared with the control group, FPG of the two exercise groups had significantly decreased from the third month of intervention (p < 0.05). HbA1c of the AE group was significantly lower than that of the control group at the third month of intervention (d = -0.712, p = 0.017), while HbA1c of the BFR-RT group was significantly lower than that of the control group at the sixth month of intervention (d = -0.645, p = 0.032). Compared with baseline, the FPG and HbA1c of the two exercise groups had significantly decreased at the third month of intervention (p < 0.05) and maintained until the end of the intervention. At the sixth month of intervention, the decrease in FPG [-0.53 (-0.89, -0.16)] and HbA1c [-0.52 (-0.64, -0.40)] in the AE group was greater than FPG [-0.46 (-0.81, -0.10)] and HbA1c [-0.44 (-0.55, -0.32)] in the BFR-RT group, but there were no significant differences in FPG and HbA1c among the two exercise groups (p > 0.05).

### 3.6 Lipid profiles

**Table 4** shows the changes in the lipid profiles of the three groups of participants. There were no significant differences in the lipid parameters among the three groups at baseline (p > 0.05). Compared with the control group, at the third month of intervention, the TC (d = -0.679, p = 0.013), TG (d = -0.632, p = 0.033), and ApoB (d = -0.812, p = 0.007) in the AE group had significantly decreased, while the ApoA1 (d = 0.858, p = 0.007) in the BFR-RT group had significantly increased, and the LDL-C, non-HDL-C, and ApoB/ApoA1 ratio in both exercise groups had significantly decreased (p < 0.05). At the sixth month of intervention, the TC, TG, LDL-C, HDL-C, non-HDL-C, ApoA1, ApoB, and ApoB/ApoA1 ratio in both exercise groups had significantly improved compared with the control group (p < 0.05). During the intervention period, there were no significant differences in lipoprotein (a) among the three groups (p > 0.05). Compared with baseline, from the third month of intervention, the TC, TG, LDL-C, HDL-C, non-HDL-C, ApoA1, ApoB, and ApoB/ApoA1 ratio in both exercise groups had significantly improved, and these improvements remained significant until the sixth month of intervention. The lipoprotein (a) in the BFR-RT group had significantly decreased compared with baseline at the third month of intervention [-4.59 (-9.13, -0.05), p = 0.046], and in the sixth month of intervention, the lipoprotein (a) in the AE group was significantly lower than baseline. At the sixth month of intervention, the HDL-C, ApoB, and ApoB/ApoA1 ratio in the control group had significantly increased compared with baseline (p < 0.05), while the remaining parameters of lipid in the control group showed no significant differences before and after intervention (p > 0.05). There were no significant differences in the lipid profiles between the two exercise groups (p > 0.05).

### 3.7 SBP and DBP

**Table 5** shows the changes in BP among the three groups of participants. At baseline, there was no significant difference in BP among the three groups (p > 0.05). From the third month of the intervention, the SBP in both exercise groups was significantly lower than that in the control group and at baseline (p < 0.05). Compared with baseline, there was no significant change in BP in the control group (p > 0.05). The results of the repeated measures analysis of variance showed that the interaction effect between group and time (p = 0.126) and the main effect of group (p = 0.588) had no significant effect on the DBP of the three groups, while the main effect of time had a significant effect on DBP (p = 0.005). At the sixth month of intervention, the DBP in the AE group decreased significantly compared with baseline [-2.90(-5.42, -0.38), p = 0.018], and the DBP in the BFR-RT group also decreased, but the difference was not significant compared with baseline [-2.19(-4.63, -0.25), p = 0.094].

### 3.8 Obesity indicators

**Table 6** shows the changes in WHtR, BMI, and TyG among the three groups of participants. At baseline, there were no significant differences in WHtR, BMI, and TyG among the three groups (p > 0.05). Both exercise groups showed significant decreases in WHtR, BMI, and TyG compared with the control group and baseline from the third month of intervention (p < 0.05), and the decrease was even more pronounced at the sixth month of intervention (p < 0.001). The BMI decrease in the AE group [-1.81 (-2.06, -1.55)] was slightly greater than that in the BFR-RT group [-1.46 (-1.70, -1.21)], but there were no significant differences in WHtR, BMI, and TyG between the two exercise groups (p > 0.05). Compared with baseline, the control group showed significant increases in WHtR and BMI from the third month of follow-up (p < 0.05).

### 3.9 Safety outcomes

Before randomization, the researchers evaluated the physical condition of the participants and provided necessary protection and monitoring during the intervention. Therefore, there were no serious adverse events during the six-month study period. However, five adverse events were reported (control group, n=1; AE, n=2; BFR-RT, n=2). The control group reported that a participant suddenly felt dizzy, palpitations, sweating, and shortness of breath while exercising at home. After resting for a few minutes, the condition improved spontaneously. After consultation, it was mainly due to the patient’s previous lack of exercise habits, insufficient warm-up before exercise, and excessive exercise intensity in a short period of time. The AE group reported that a patient felt hungry at home after exercise but did not eat in time, and then had a hypoglycemic reaction. The blood glucose was measured at 3.8 mmol/L at that time, and the patient was conscious. The symptoms alleviated after immediate eating. One participant in the AE group withdrew from the study due to recurrent joint pain after one month of exercise. The BFR-RT group reported that a patient’s blood glucose was measured at 4.5 mmol/L two hours after dinner before exercise, with no complaints of discomfort. The researchers advised the patient to rest after eating soda crackers, but the patient insisted on participating in the training. After 15 minutes of exercise, the blood glucose was measured at 4.3 mmol/L. To prevent hypoglycemia, the researchers advised the patient to rest and have some bread until the blood glucose was greater than 5.6 mmol/L. The patient reduced the dose of antidiabetic drugs according to medical advice and did not show a tendency towards hypoglycemia during the study period. Another participant in the BFR-RT group had a few subcutaneous bruises on the skin around the air band compression site during the first training, but there was no pain or other discomfort symptoms. After reducing the pressure of the air band to 40% LOP and ensuring that the clothing was smooth, the bruises disappeared.

## 4 Discussion

**Figure 4:**
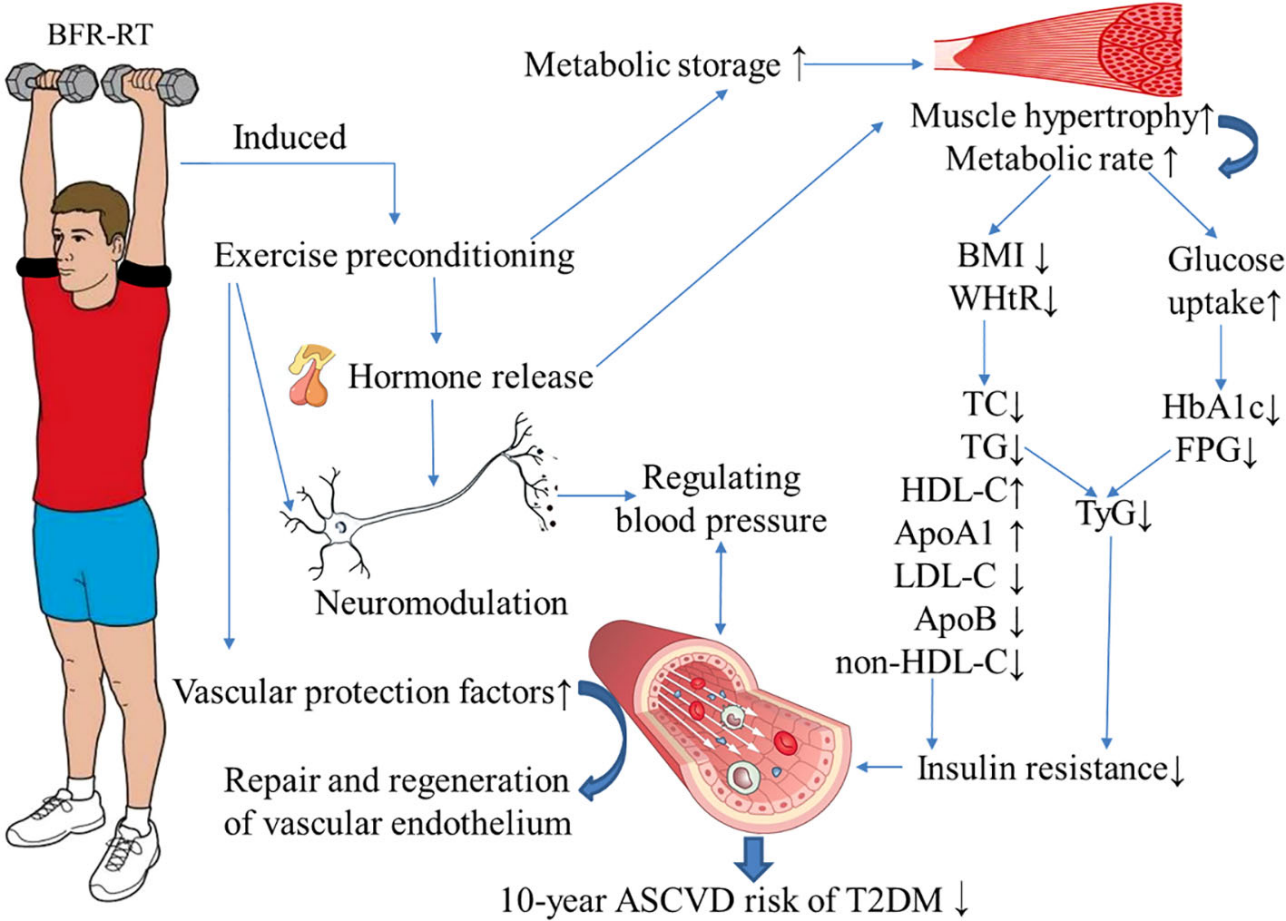
Possible mechanism of blood flow-restrictive resistance training (BFR-RT) improving the 10-year risk of atherosclerotic disease (ASCVD) in patients with type 2 diabetes (T2DM). During BFR-RT, the intermittent restriction of blood flow in the limbs simulates the process of tissue ischemia/reperfusion, inducing exercise preconditioning. This process prompts the production, activation, and release of certain hormones (such as growth hormone and insulin-like growth factor-1), metabolites (such as lactic acid), cytokines, and free radicals (such as nitric oxide) into the bloodstream within a short period of time. This leads to hypertrophy of muscle tissue and an increase in metabolic rate. The increased muscle fibers enhance the uptake and utilization of glucose and lipids, leading to a decrease in fasting plasma glucose (FPG) and glycosylated hemoglobin (HbA1c) in patients with T2DM. Additionally, by reducing the body mass index (BMI) and waist-to-height ratio (WHtR), it helps control obesity, thereby improving dyslipidemia and reducing insulin resistance in patients with T2DM. Long-term BFR-RT can also regulate blood pressure through neurohumoral pathways, stimulate the release of vascular protective factors, promote the repair and regeneration of vascular endothelium, and reduce the 10-year ASCVD risk in middle-aged T2DM patients. TC, total cholesterol; TG, triglycerides; HDL-C, high-density lipoprotein cholesterol; LDL-C, low-density lipoprotein cholesterol; ApoA1, ApolipoproteinA1; ApoB, ApolipoproteinB; TyG, triglyceride-glucose index. The blue arrow indicates the impact of BFR-RT.

This study conducted a six-month supervised exercise intervention for middle-aged patients with T2DM, and the main finding was that BFR-RT could improve the FPG, HbA1c, blood lipids, BP, and obesity indicators of middle-aged patients with T2DM, and reduce the 10-year ASCVD risk index and risk level of patients. (The specific mechanism is shown in **Figure 4**) The cardiovascular protective effect of BFR-RT on T2DM patients was similar to that of moderate-intensity AE, and no significant increase in exercise risk was found. The application of BFR-RT provides a new choice for exercise intervention in patients with T2DM.

The results of this study showed that BFR-RT and AE had similar effects in reducing FPG and HbA1c in patients with T2DM. This is consistent with the findings of Christiansen et al. They (31) conducted a 6-week single-leg BFR-RT on young men, and the results showed that the muscle glucose uptake during exercise in the thighs receiving BFR-RT was higher than before training. This may be because BFR-RT promotes muscle hypertrophy and increases muscle glucose uptake and utilization. BFR-RT can significantly increase the 5’AMP-activated protein kinase (AMPK) in muscle cells through exercise, which enhances the synthesis and expression of glucose transporter 4, accelerating the uptake of glucose and storage of glycogen in muscle cells (32). In addition, during BFR-RT, muscle ischemia activates the sympathetic nervous system, triggering the release of various energy substrates into the bloodstream. This can promote the absorption and oxidation of free fatty acids and glucose by skeletal muscle cells, enhance mitochondrial biosynthesis, and increase muscle glucose oxidation capacity. This in turn enhances the insulin sensitivity of skeletal muscle cells and improves insulin resistance in patients with T2DM (33, 34). It is worth noting that although there was no significant difference in the hypoglycemic effect between the two exercise groups, we observed that the rate and magnitude of HbA1c decline in the BFR-RT group were lower than those in the AE group. We speculate that this may be due to the increased release of multiple hormones after BFR-RT compared to non-BFR-RT (35), including growth hormone, insulin-like growth factor-1, sex hormones, and renin, etc. (36). These hormones may have a certain impact on the regulation of blood glucose, but further research is still needed to confirm this.

This study showed that BFR-RT can help improve dyslipidemia in middle-aged patients with T2DM. This is similar to some previous research results. Sun’s (37) Mata analysis included five studies with a total of 66 participants, and the results showed that BFR-RT can significantly reduce TC, TG, LDL-C, and increase HDL-C in overweight or obese adults. The possible mechanism is that BFR-RT can significantly increase the key protein PGC-1α2 that inhibits cholesterol biosynthesis *in vivo*, thereby reducing TC and LDL-C levels (38). ApoA1 mediates the reverse cholesterol transport of HDL, which can transport excess cholesterol from arterial wall cells to the liver for recycling or excretion as bile acid. This is the main mechanism of HDL in anti-atherosclerosis (39). However, the long-term high-glucose environment in patients with T2DM changes the secondary structure of ApoA1, resulting in a decrease in the cholesterol efflux ability of HDL (40). Our study showed that BFR-RT can increase the ApoA1 in middle-aged T2DM patients, reduce non-HDL-C, ApoB, and ApoB/ApoA1 ratio, and thus achieve the goal of anti-atherosclerosis. Previous studies have shown that moderate-intensity AE can increase ApoA1 in obese adolescents (41), while in our study, the time of ApoA1 elevation in the BFR-RT group was earlier than that in the AE group, indicating that BFR-RT has certain advantages in improving ApoA1 in middle-aged T2DM patients. The possible reason is that BFR-RT can cause transient ischemia/reperfusion in local tissues, generate superoxide radicals, and cause oxidative stress reactions, resulting in differential expression of ApoA1, which can better resist possible subsequent ischemic injury (42). In addition, we also found that although six months of BFR-RT significantly reduced the level of lipoprotein (a) in patients with T2DM compared to baseline, there was no significant difference compared to the control group. Kadoglou et al. (43) also showed that high-intensity (60-80% 1-RM) resistance exercise for three months had no significant effect on lipoprotein (a). This may be due to the fact that lipoprotein (a) is related to factors such as race and genetics, and exercise and diet may not have a significant impact on it. It is worth noting that although there was no difference between the two types of exercise in this study, the improvement in various components of blood lipids by BFE-RT was greater than that of AE. Previously, there were few studies comparing the effects of AE and BFR-RT on improving the lipid profile in patients with T2DM. At present, it seems that BFR-RT may have more advantages, but more research is needed to demonstrate and explore its specific mechanisms.

Over 80% of the participants in this study were obese to varying degrees, and insulin resistance caused by obesity is an important pathway for the development of ASCVD in patients with T2DM (44). This study showed that from the third month of intervention, the TyG, BMI, and WHtR of the two exercise groups had significantly decreased compared to the control group and baseline, indicating that AE and BFR-RT can effectively reduce the waist circumference and body weight of patients with T2DM, control insulin resistance, and thereby reduce the risk of ASCVD. In addition, we also observed that the decrease in BMI in the BFR-RT group was less than that in the AE group, which may be due to the increase in muscle mass in participants due to BFR-RT. When the body’s muscle content increases, energy consumption at rest also increases, which helps improve lipid metabolism, reduce the accumulation of fat in the liver and muscles, and thus reduce insulin resistance.

The results of this study showed that after the intervention, SBP in both exercise groups decreased significantly, but DBP did not decrease significantly, which may be related to the participants’ baseline DBP being normal, and suggests that both exercises have a good regulatory effect on BP and do not cause abnormal hypotensive reactions. In previous studies, the effect of BFR-RT on the BP has been controversial. Maior et al. (45) showed that young men who performed a single high-intensity (80% 1-RM) resistance exercise and low-intensity (20-40% 1-RM) BFR-RT both significantly reduced SBP and DBP 30 minutes after training compared to baseline, with no significantly difference between the two exercises. Araújo et al. (46) pointed out that BFR-RT is more effective than moderate-intensity (50%1-RM) resistance training in reducing SBP in middle-aged hypertensive patients. However, Scott et al. (47) pointed out that the SBP, DBP and mean arterial pressure of elderly women after BFR-RT were higher than those of high-intensity (70% 1-RM) resistance training. BFR-RT decreases the blood perfusion of exercising muscles, which may activate the ergoreflex (i.e. the metaboreflex and the mechanoreflex), especially in the patients of cardiovascular underlying diseases, leading to excessive sympathetic activity, and resulting in elevated BP and other CVD events. Even healthy individuals may face the risk (48). Therefore, researchers were called upon to focus on safety before using BFR-RT to obtain cardiovascular protection benefits (49). During BFR-RT, the higher cuff pressure, the longer pressurization time, and the greater exercise load, the stronger cardiovascular and sensory (such as fatigue, muscle soreness, etc.) responses will be (50–52). Selecting 40% LOP for BFR-RT not only provides ischemic stimulation comparable to 80% LOP, but also reduces cardiovascular response (53). intermittent blood flow restriction can better improve muscle performance and reduce muscle fatigue than continuous blood flow restriction (54). In view of this, we developed the BFR-RT programme, the results showed that the BFR-RT achieved a similar antihypertensive effect to AE, and no serious adverse events occurred during the study period. This may be because long-term regular BFR-RT can improve vascular compliance, blunt ergoreflex sensitivity, restore the balance of sympathetic-vagal reflex in T2DM patients (55).

This study showed that from the third month of intervention, the 10-year ASCVD risk index of the two exercise groups had significantly decreased. This indicates that BFR-RT, like AE, can effectively improve abnormal glucose metabolism, dyslipidemia, hypertension, and obesity to reduce the risk of ASCVD in middle-aged T2DM patients. It is worth noting that although there was no significant difference between the two types of exercise in reducing the risk level of ASCVD, the speed of BFR-RT seems to be faster. This may be because long-term BFR-RT can also induce anti-atherosclerotic cardiovascular adaptation (56). During BFR-RT, the process of cuff compression and relaxation on the limb induces exercise preconditioning, promoting the production, activation, and release of some hormones, metabolites (such as lactic acid), cytokines, free radicals (such as nitric oxide), and other substances into the bloodstream in a short period of time. These substances are transported by the bloodstream to multiple organs, including the heart, and can help tissues and organs resist subsequent damage caused by prolonged ischemia and hypoxia (57). BFR-RT increases lactate in the body, promotes myocardial cell metabolism, and upregulates the expression of microRNAs associated with arterial formation (58). BFR-RT enhances ischemia and intravascular shear stress, promotes the synthesis and bioavailability of nitric oxide in vascular endothelium, and improves vascular endothelium-dependent relaxation function (59). BFR-RT can also stimulate the proliferation and differentiation of vascular endothelial progenitor cells, increase the activity of early markers of cardiovascular health, and promote the repair and regeneration of vascular endothelium (38). The improvement of vascular endothelial integrity and function by BFR-RT helps to slow the progression of ASCVD, and adherence to exercise can maintain this protective effect (60), making it an attractive option for T2DM patients who cannot tolerate moderate to high-intensity exercise.

## 5 Limitations of the study

The limitation of this study was that we did not provide an accurate daily energy intake data for individuals, as the intervention we provided was not conducted in a fully enclosed state, and could not provide three meals a day with the same caloric intake. Instead, we supported participants to maintain some lifestyle habits according to their own wishes. And Chinese people are used to eating several different dishes with family members at a meal, and the food has been cooked in different ways, so it is difficult to estimate the calories in the food by weighing it, which may affect our interpretation of some data. Although our research results confirmed that BFR-RT is safe for middle-aged T2DM patients and there were no serious adverse events such as thrombosis, stroke, myocardial infarction, etc. during the study, the sample size included in our study was small, and the participants did not have serious complications or uncontrolled diseases. Currently, there is no data available for reporting and analyzing the changes in thrombus risk and inflammation-related indicators in T2DM patients after BFR-RT. Therefore, there is not enough evidence to prove the effectiveness and safety of BFR-RT for patients with diabetes complications such as diabetic nephropathy, diabetic foot, and neuropathy. It is expected that further research will reveal these findings.

## 6 Conclusion

In summary, low-intensity, intermittent BFR-RT can significantly increase muscle glucose uptake and utilization, reduce blood glucose levels, improve insulin sensitivity, regulate blood lipids and BP, control obesity, and induce protective cardiovascular adaptation, thereby reducing the 10-year ASCVD risk in middle-aged T2DM patients. Patients with T2DM can use BFR-RT as an alternative to moderate-intensity AE according to individual circumstances to achieve early exercise adaptation and cardiovascular benefits. However, it is worth noting that our research on BFR-RT is still in the preliminary exploratory stage, and it is uncertain whether BFR-RT has the same protective effect on people who have already developed diabetic vascular complications. To ensure safety, it is recommended that patients with T2DM undergo adequate medical screening and CVD risk assessment before undergoing BFR-RT, and do so under the guidance of a professional.

## Data Availability

The original contributions presented in the study are included in the article/supplementary material. Further inquiries can be directed to the corresponding authors.
